# Persistence of functional microbiota composition across generations

**DOI:** 10.1038/s41598-021-98097-3

**Published:** 2021-09-24

**Authors:** Christian Ramos, Mario Calus, Dirkjan Schokker

**Affiliations:** 1 Animal Breeding and Genomics, Wageningen University & Research, P.O. Box 338, 6700 AH Wageningen, The Netherlands; 2 Carrera de Biología, Facultad de Ciencias Puras Y Naturales, Universidad Mayor de San Andrés, Casilla 10077—Correo Central, La Paz, Bolivia

**Keywords:** Microbiology, Evolution

## Abstract

Holobionts are defined as a host and its microbiota, however, only a fraction of the bacteria are inherited vertically and thus coevolve with the host. The “it’s the song, not the singer” theory proposes that functional traits, instead of taxonomical microbiota composition, could be preserved across generations if interspecies interaction patterns perpetuate themselves. We tested conservation of functional composition across generations using zooplankton, mosquito, and plant datasets. Then, we tested if there is a change of functional microbiota composition over time within a generation in human datasets. Finally, we simulated microbiota communities to investigate if (pairwise) interactions can lead to multiple stable community compositions. Our results suggest that the vertically transmitted microbiota starts a predictable change of functions performed by the microbiota over time, whose robustness depends on the arrival of diverse migrants. This succession culminates in a stable functional composition state. The results suggest that the host-microbiota interaction and higher order interactions in general have an important contribution to the robustness of the final community. If the proposed mechanism proves to be valid for a diverse array of host species, this would support the concept of holobionts being used as units of selection, including animal breeding, suggesting this has a wider applicability.

## Introduction

A holobiont is defined as the combination of an organism, also called host, with its microbiota^1,2^. The microbiome, which term we here use to refer to all the microbiota carried by a host (gut, skin, leaf, etc.)^3^, affects the phenotype of a host, and that effect is different from the host genotype or its environment^4^. The microbiota composition is at least partially transmitted across generations, which makes it a non-genetic form of inheritance^4^. Non-genetic forms of inheritance are not yet incorporated into breeding strategies^5^. Hence, to understand how the microbiota is assembled within a host, and then inherited across generations, could increase the progress of animal breeding programs.

The mechanisms of symbiont microbiota inheritance and persistence vary across species of hosts and symbionts^6^. This phenomenon is observed throughout evolution, in the animal kingdom for instance, squid (*Euprymna scolopes*) parents release the bacteria *Vibrio fischeri* to open water and by that way transmitting the bacteria^7^; juvenile koalas get tannin degrading bacteria eating enriched maternal faeces called ‘pap’^8^; in chickens, bacteria from the cloaca and oviduct colonize the egg shell, egg white and from there, the embryo^9,10^; in livestock, new-borns are initially colonized through physical contact when passing through the birth canal^4^; within the plant kingdom, some plants transmit specific bacteria and/or fungi via vegetative reproduction^11^. Such transmission systems can be classified as vertical (from one generation to the next), horizontal (within generations), or a combination of both^12^.

After the arrival of a specific symbiont to a host, its extinction or survival depends on the interactions with the host and the resident microbiota, and whether or not the host environment acts as a filter^12^. The host regulates the microbiota composition through a constant crosstalk that involves its innate and adaptive immune system as well as the epithelial cells in direct contact with symbionts^13^. The interactions or lack thereof between symbionts are typically described with competition models borrowed from ecology, that incorporate or omit time structure^14^. For instance, the generalized Lotka-Volterra models use a matrix to represent the interactions between every pair of species in a community (pairwise species interactions) and describe the population dynamics over time of each species with a differential equation^14^. The self-organized instability (SOI) model also describes interspecies interactions using a matrix, but the population dynamics are stochastic and represented with a mechanistic set of rules that allows the simulation of a community^15^. Such descriptions do not capture higher order interactions nor account for the migration between hosts^12^. The process of maturation of a community within a host sometimes displays clear sequences, such as the transition from facultative to strict anaerobic bacteria in humans^16^; this process is analogous to an ecological succession, which in this case leads to a stable community^17,18^.

Whether or not the concept of holobionts is needed or redundant is still debated^2,4,12,19–21^. Given the diverse array of symbionts within a host, selection at the holobiont level cannot account for all the host-symbiont interactions, nor the interactions between symbionts, as the requirements for partner fidelity are unlikely to be met for every interaction^20^. Yet, there are developmental, physiological, anatomical, and immunological traits that rely on host-symbiont interactions^12^. Given that the microbiota shows redundance at the functional level^22,23^; Doolittle and Booth^19^ proposed that interspecies interaction patterns can perpetuate themselves over time through “recurrence” (as opposed to replication), regardless of the specific species that form part of the interaction. The mechanism of mutual perpetuation between the interaction patterns and the specific traits of the interacting species is called the “It’s the song, not the singer” (ITSNTS) theory^24^, where their focus is on novel mechanisms of recurrence across generations.

**Figure 1:**
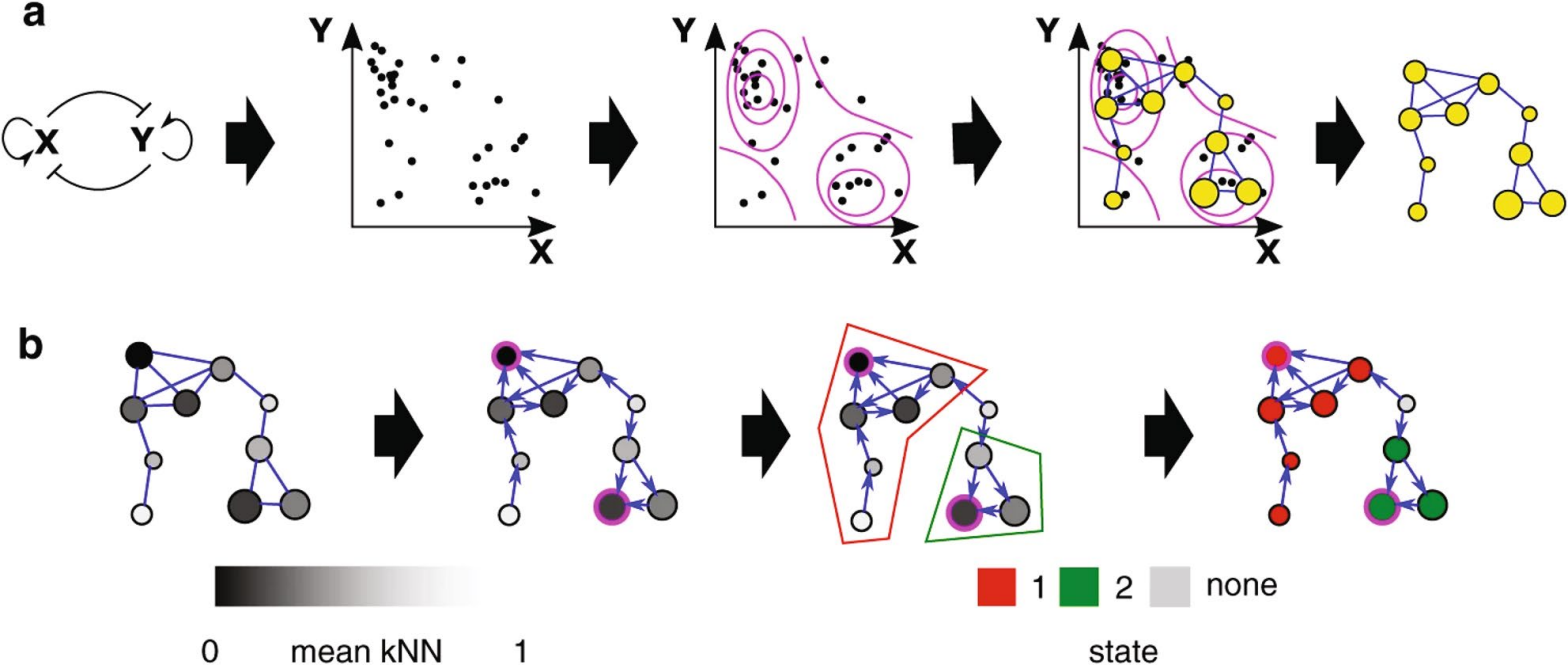
Microbial communities as metastable dynamical systems. This illustration of the Mapper algorithm is adopted without modifications from^25^ and is licensed under CC BY 4.0. (**a**) A community with two species X and Y, that inhibit each other’s growth. The interactions between species make some community compositions more likely than others: either one species is very abundant at the cost of the other, or vice versa. If those configurations are equilibrium states and stable against small perturbations (i.e., if they attract all configurations in their vicinity), they can be considered attractors. The mapper plot can be used to find attractors: It groups samples into clusters (yellow dots) according to their similarity. Clusters with similar compositions are plotted close to each other. The same data-point (a sampled community) can belong to more than one cluster. Then, it connects the clusters that share communities (samples) making a network. Because clusters have different numbers of samples, those with the highest local number of samples potentially are attractors. (**b**) Finding the attractors in the mapper network. Each node is assigned a k-nearest neighbours (kNN) value (inversely proportional to the density of points) that is later modified to get a potential. The arrows are then directed from higher to lower potential. The nodes with the lowest potentials correspond to attractors, and the nodes that lead to them form their corresponding basin of attraction (all the community states that eventually converge to the attractor). The outcome shows two attractors that correspond to either X or Y with high abundance and the other excluded.

The ITSNTS theory applied to holobionts^19^ assumes that the interaction patterns within a single generation remain constant across time, but it is possible to extend the idea through a developmental approach. Chang et al.^25^ and Shaw et al.^26^ showed that if we consider microbiota as a dynamical system, then the communities display metastability: there are several possible discrete stable configurations, that correspond to alternative late succession communities. For instance, coral reefs can display bi-stability^27^ and semiarid/arid communities are two configurations of a bi-stable system that depends on the aridity level^28^. The coexistence of alternative late successional states is also displayed by ecological systems with asymmetrical competition^29^ and by metacommunities far from equilibrium (steady state) or with variation in local community quality or species traits^30^. Given that the attractor (i.e. a stable discrete configuration) reached by a community depends on the initial state, as shown e.g. for coral reefs^27^, metastability combined with the aforementioned ecological succession could account for the conservation of functional composition (the biochemical functions performed by the symbionts) across generations predicted by Doolittle & Booth^2^: The conserved functional composition would correspond to an attractor, which is stable because of the interaction patterns (Fig. 1a). The vertically inherited fraction of the community could define a starting configuration, from which the community converges to a specific attractor in a functional space, regardless of the taxa of the migrants.

Therefore, our objectives were to investigate to what extent functional composition is preserved across generations in experimental lab conditions (1), and to propose a mechanism for this preservation based on the ITSNTS theory. For the latter, we tested if there is an ecological succession functional space, and if it depends on the initial community composition (2); we then searched for metastability in the functional composition of communities (3); and tested if pairwise interactions can account for that metastability (4).

These objectives were addressed by investigating different publicly available datasets that matched the requirements for one or more of those objectives. For (1), we compared the functional composition of zooplankton, mosquito, and plant microbiota across generations. For (2), we compared the functional composition of monthly faecal samples from babies born either vaginally or with caesarean section. All datasets can display metastability and are used for (3). We simulated communities based on stochastic mechanistic models for (4). Whereas (1) tests directly functional recurrence in holobionts, (2) and (3) together test a possible mechanism of transmission across generations and (4) narrows down the set of conditions needed by that mechanism.

## Methods

### Datasets

To test functional persistence across generations, we used the zooplankton dataset from^31^, the mosquito dataset from^32^, and the plant dataset from^11^ (Table 1). The first dataset comprises of five generations where offspring of the control group was placed in the control group, and offspring of the antibiotic treatment to remain in the antibiotic group or recover in the control group (leading to a total of four recovery groups). The second dataset comprises generations 0, 5 and 10 of two isolated mosquito populations bred with lab or field water, originally used to test if the microbiota is preserved or not under different lab breeding conditions (with the different water sources). The third dataset comprises generations 0, 1 and 2 of plants cloned via stolons used to infer a core set of taxa transmitted vertically common to 10 representative ecotypes of the species. Hence, horizontal transmission was completely inhibited and both roots and stolons were sampled.

**Table 1:** Datasets included in this study.

Species	Zooplankton (*Daphnia magna*)	Mosquito (*Anopheles gambiae*)	Plant (*Glechoma hederacea*)	Human (*Homo sapiens*)	Human (*Homo sapiens*)
Generations	1; 2; 3; 4; 5	0; 5; 10	1; 2; 3	*	*
Number of individuals	4 per treatment**	13***	50	43	212
Sample number	83	52	90	920	765
Sample type	16 s	16 s	16 s/18 s	16 s	Whole Metagenome
Accession number	PRJNA703930	doi:10.5061/ dryad.98jj7gk	PRJEB20603	PRJEB14529	PRJNA290380
Database	ENA	Dryad	ENA	ENA	ENA
Reference	^29^	^30^	^11^	^31^	^32^

*All the samples belong to a single generation, ** 5 individuals were pooled, *** Number of treatment replicates instead of individuals.

To test the ecological succession on the functional landscape, we used the human datasets of^33,34^. The first one was originally used to describe how different perturbations (antibiotics, birth mode, diet) affect the early community development and the second one is part of the DIABIMMUNE project, aimed at testing the role of the microbiota in the development of autoimmune diseases. These two human datasets comprise the faecal microbiota of approximately 40 and 200 human babies (Table 1) repeatedly sampled from birth and up to three years, born either naturally or with a caesarean section.

### Data processing and analysis

All the raw samples were processed using the DADA2 pipeline^35^. The human samples from^33^ were truncated at 150 bp, the forward and reverse mosquito samples were truncated at 250 and 200 bp respectively and both forward and reverse plant samples were truncated at 250 bp. Every other filter and trimming parameter was kept at the standard configuration for all samples (maxN = 0, maxEE = 2, truncQ = 2, rm.phix = TRUE). The unique sequences were inferred without pooling across samples. The corresponding amplicon sequence variant (ASV) tables were functionally annotated using Tax4Fun2 using the default reference database and parameters^36^. The second human dataset was already processed through the MGnify pipeline^37^ version 4.1^38^, and the Gene Ontology (GO) composition of each community was used directly.

### Landscape analysis of the functional composition

The functional predictions were then used to run the landscape model^25^. The frequencies of the predicted functions, instead of the frequencies of ASVs, were used to calculate the Jensen Shannon divergence between communities. The resulting dissimilarity matrices were then used to run a Principal Coordinate Analysis (PCoA). The two first Principal Coordinates (PCos) were used as the filter functions for the Mapper algorithm (Fig. 1a)^39^, moreover we calculated the percentage of variance explained by the PCos (Table 2) for all datasets. The Mapper algorithm was performed with the following hyperparameters (kept exactly as in^25^) for all datasets: Number of intervals for rank = 15 for both PCos, % overlap = 70% and number of bins = 10. Finally, the mapper output was transformed in a directed graph to find the attractors and corresponding basins (Fig. 1b)^25^. We are running the Mapper algorithm to find attractors in the functional space, and therefore different communities that perform the same functions will belong to the same attractor. Hence, the conservation of the attractors through generations is an indirect test of functional recurrence.

**Table 2:** Variance explained* by the first 10 Principal Coordinates (PCos).

PCo	Zooplankton^29^	Mosquito^30^	Plant^11^	Human^31^	Human^32^
1	27.71	56.48	42.82	39.88	31.31
2	22.82	22.65	19.44	15.65	18.90
3	9.88	5.48	7.99	10.94	8.35
4	8.35	4.38	6.25	4.87	3.15
5	7.42	3.90	3.00	2.64	2.73
6	6.68	2.29	2.77	2.32	1.78
7	5.65	1.89	2.56	1.88	1.54
8	2.10	0.90	2.20	1.52	1.13
9	1.58	0.55	1.61	1.46	1.03
10	1.34	0.46	1.27	1.19	0.91
Total	**93.52**	**98.98**	**89.91**	**82.35**	**70.82**

*Relative eigenvalues.

### Simulations of the Hubbell and SOI model

Simulations were run to test if pairwise interactions can account for metastability in the taxonomical community composition. The communities were simulated using either the SOI model^15^ or the Hubbell model^40^ using the R-package seqtime^14^. While the Hubbell model simulates communities without pairwise interactions, the SOI model includes the fraction of all the possible pairwise interactions as a parameter (connectivity). The chosen connectivity values were 0 (the Hubbell model), 0.01 (as used in the simulations of^14^), and 0.1 (as used in^15^). In addition, a fraction of individuals (hosts) from the initial composition was fixed: every simulation started with a randomly chosen and a fixed group of individuals.

Either 0, 100, or 200 of all 500 simulated individuals were fixed. 450 replicates were simulated for every parameter combination. All replicates from a given parameter combination shares the interaction matrix, the initial fixed individuals, and the migration probabilities. When generating the interaction matrices, the positive edge percentage (the percentage of non 0 values of the interaction matrix that are greater than 0) was kept at most at 30%, switching randomly the signs of the matrix elements^14^. All communities were simulated for 600 timesteps. For all the communities the metacommunity species number was kept at 50, the migration probability per species was drawn from a standard uniform distribution.

The similarity between pairs of communities with the same parameters was measured using the Morisita index (which decreases with higher beta diversity and approaching 0 for completely different communities)^41^. From the 450 replicates per treatment, we grouped them in pairs, and each community was used only once, resulting in 225 pairs. The effect of the connectivity, the number of fixed individuals and the interaction between both on the Morisita index was tested using a generalized linear model with an inverse link and a gamma distribution, using the package *stats* from base R:

$${y}_{ijk}={g}^{-1}\left({\beta }_{0}+{\beta }_{1}{c}_{i}+{\beta }_{2}{x}_{j}+{\beta }_{3}c{x}_{ij}+{e}_{ijk}\right)$$

where ${c}_{i}$ is the ${i}{th}$ connectivity value (0, 0.01 or 0.1), ${x}_{j}$ is the ${j}{th}$ number of individuals (0, 100 or 200) fixed at the start of the simulation, $c{x}_{ij}$ is the interaction term and ${y}_{ijk}$ is the Morisita index of the ${k}{th}$ replicate. The main and nested models were ranked according to their Akaike information criterion corrected for small samples (AICc), using the MuMIn package^41^. That is, we ranked them based on how well they fit the data, according to their log-likelihood given the data, while penalizing models with more parameters.

## Results

### Persistence of functional traits across generations

**Figure 2:**
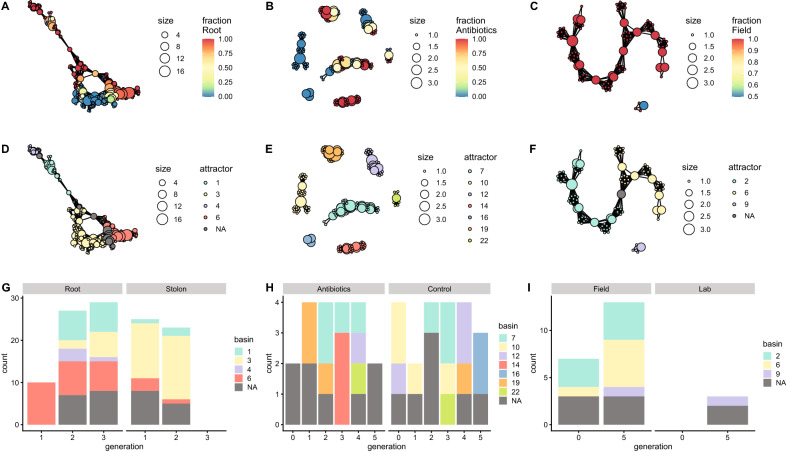
Persistence of the functional composition of plant, zooplankton, and mosquito microbiota across generations. (**A**) Mapper representation of root and stolon plant samples. The colour represents the fraction of root samples per node. (**B**) Mapper representation of the zooplankton samples. The colour represents the fraction of samples from zooplankton communities bred in the lab as opposed to antibiotics in the first generation. (**C**) Mapper representation of the mosquito samples. The colour represents the fraction of samples from mosquito communities bred in field water as opposed to lab water. (**D**) Partition of the plant mapper network into metastable states. Each colour represents a different attractor. (**E**) Partition of the zooplankton mapper network into metastable states. Each colour represents a different attractor. (**F**) Partition of the mosquito mapper network into metastable states. Each colour represents a different attractor. (**G**) Count of plant samples that belong to each attractor, per generation and tissue. R = Root and S = Stolon. (**H**) Count of zooplankton samples that belong to each attractor, per generation and treatment. (**I**) Count of mosquito samples that belong to each attractor, per generation and treatment.

Results from the plant, zooplankton, and mosquito mapper plots show metastability (Fig. 2A–C), i.e. there are groups of nodes with different colours, and each colour represents a different attractor (Fig. 2D–F). Additionally, populations show an increase in the diversity of occupied attractors across generations (Fig. 2G–I), even when accounting for the plant tissue, antibiotics, or the water type, respectively (Fig. 2D–I). The antibiotics treated zooplankton in generation 1 resulted in a single attractor in the fifth generation compared to two attractors for the zooplankton in the control group (Fig. 2E,H). The lab water-bred mosquitoes belong to a single attractor, or do not belong to an attractor at all (Fig. 2F,I). There are fewer counts on generation 5 of lab water mosquitoes and not a single count from generation 10 mosquitoes, because every corresponding vertex is a singleton (has a single sample) and thus is removed from the mapper plot. The increase of diversity of occupied attractors and the increase of singletons in the datasets imply that the functional composition is not preserved across generations. The populations occupy discrete attractors, but the attractors are different each generation.

### Ecological succession in the functional space

**Figure 3:**
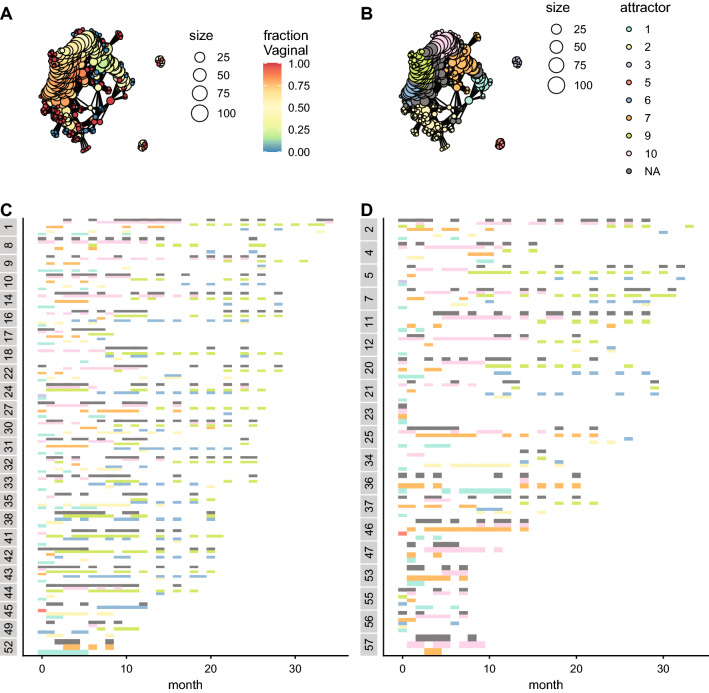
Ecological succession of human microbiota communities in a functional phase space, using the KEGG annotation. (**A**) Mapper representation of the samples. The colour represents the fraction of samples from individuals born vaginally, instead of caesarean section. (**B**) Partition of the mapper network into metastable states. Each colour corresponds to an attractor. (**C**,**D**) Attractor occupied per individual and month. Each row corresponds to a single individual. The colours correspond to the attractors in (**B**). (**C**) Individuals born vaginally. (**D**) Individuals born via caesarean section.

**Figure 4:**
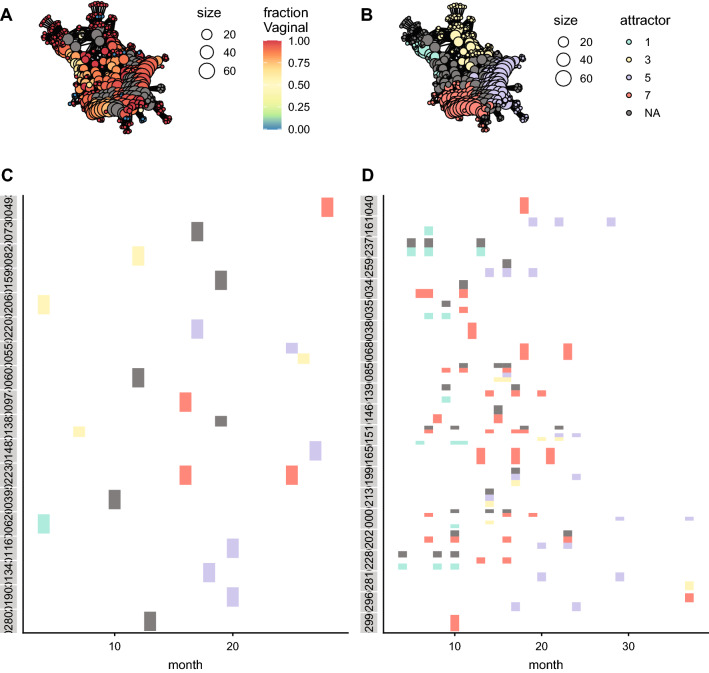
Ecological succession of human microbiota communities in a functional phase space, using the GO annotation. (**A**) Mapper representation of the samples. The colour represents the fraction of samples from individuals born vaginally, instead of caesarean section. (**B**) Partition of the mapper network into metastable states. Each colour corresponds to an attractor. (**C**,**D**) Attractor occupied per individual and month. Each row corresponds to a single individual. The colours correspond to the attractors in (**B**). (**C**) Individuals born vaginally. (**D**) Individuals born via caesarean section.

After comparing the functional composition across generations, we tested if there is a successional pattern in the human datasets within a generation. The mapper algorithm also splits the mapper network into attractors for both datasets (Figs. 3B and 4B); but, given that the samples only span the first three years of life of every subject, those attractors are only transiently occupied. All the samples converge to the attractors, regardless of the source of the microbiota (vaginal or caesarean section) (Figs. 3A,B and 4A,B). There are clear trajectories in the functional space across time that are independent of the source of the microbiota (Figs. 3C,D and 4C,D).

### Intraspecies interactions as a source of metastability

**Figure 5:**
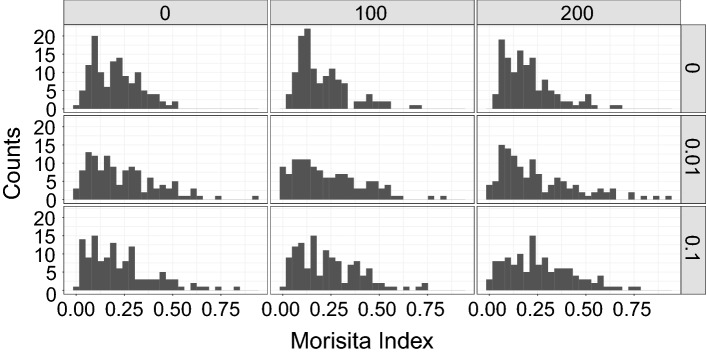
Histograms of the Morisita dissimilarity index for pairs of simulated communities. Rows: fraction of the connectivity matrix filled with non-zero values (see methods). Columns: Number of fixed shared individuals (out of a total of 500) at the start of each simulation.

Having found evidence suggesting metastability in the plant and mosquito datasets, we simulated communities to investigate if pairwise interspecies interactions can account for it. As the amount of interactions increases, the differing starting conditions could either lead to convergence or divergence of community composition between pairs of communities. This would be reflected as a change in the Morisita index explained by the interaction between the two parameters (connectivity and initial fixed individuals). After the simulations, most Morisita index values lie between 0 and 0.75 (Fig. 5), and all are negative. Hence, the alpha (within communities) diversity is higher than the beta (between communities) diversity for all simulations, i.e. the diversity within hosts is higher than the diversity between hosts. The best model (according to the AICc ranking) only includes connectivity (Table 3) and the complete model only appears in third place; in other words, connectivity alone explains the differences in the index values. As there is not a significant difference between the best and complete models (Deviance = 1.2672, df = 2, P = 0.2853), there is no evidence that the interaction effect predicts the beta diversity. Therefore, the results of the simulations do not support metastability. In other words, the different initial conditions do not converge to a limited number of community states over time, even when there is high connectivity.

**Table 3:** Full and nested models ordered according to AICc.

Model	df	logLik	AICc	Delta	Weight
Con	3	618.674	− 1231.3	0	0.389
Con + Fix	4	619.256	− 1230.5	0.85	0.254
Con + Fix + Con:Fix	5	619.803	− 1229.6	1.77	0.16
	2	616.477	− 1228.9	2.38	0.118
Fix	3	617.058	− 1228.1	3.23	0.077

*df* degrees of freedom, *logLik* log likelihood, *AICc* Akaike Information Criterion corrected for small sample sizes, *delta* difference of AICc compared to the best model; weight: probability that the model explains the dataset; *Con* The model includes connectivity, *Fix* The model includes the number of fixed individuals at the start, *Con:Fix* The model includes the interaction term between the two independent variables.

## Discussion

The ITSNTS theory^24^ proposes that interaction patterns in the microbiome behave as units of selection. In other words, such patterns ‘recur’ across generations, there is variation between patterns, and selection can act upon those variations. Here, we tested the recurrence across generations, and we will conclude by proposing a possible mechanism. Functional annotations of metagenomic and 16S samples are used as a proxy of interactions; This assumes that the predicted functions of the genes found in the microbiota reflect the underlying metabolic networks. Our approach has two main limitations: not all the functionally annotated genes play a direct role in the interactions, and 16S samples have limited resolution. We acknowledged this by focussing on community level comparisons rather than specific taxa/functions, although an intrinsic similarity can be expected because only published genomes are used. Nevertheless, the one WGS dataset does corroborate the findings of the 16S datasets, i.e. preservation of functional composition. These afore mentioned limitations may still persist in the results and the interpretation thereof, because we used publicly available data wet-laboratory validation is very difficult to perform. However, in our study we focused on mathematical and statistical approaches of proving the underlying theory.

We carried out three different methods: first, we tested if there is conservation of microbiota functional composition across generations using zooplankton, mosquitoes and plants as model organisms. Thereafter, we explored possible mechanisms to explain this conservation across generations. Two human datasets were used to investigate if the functional composition can be understood as an ecological succession; in other words, if there is a robust change of functional composition over time, common to every host. Finally, we simulated communities to observe if pairwise interactions between bacteria can generate the metastability needed to explain the succession. This section starts with discussing the implications of the results of each of those individual analyses. Thereafter, we make a synthesis of our results, which allows to propose a mechanism that could explain the inheritance of the functional composition of microbiota across generations. We end with a brief discussion of possible implications for animal breeding.

### Conservation of functional composition across generations

Contrary to our expectations, neither plants nor mosquitoes showed conservation of functional composition across generations, thus failing to support the ITSNTS hypothesis. This could imply that there is functional recurrence across generations only when there is a wide enough metacommunity (several communities connected by migration) to draw migrants from, as zooplankton, mosquitoes, and plants were bred under conditions with decreased or even no horizontal transmission. Similarly, plant monocultures show decreased taxonomical diversity^42^, and decreased vertical and horizontal transmission is leading to cumulative microbial extinctions in humans, affecting the phenotype and development of the immune system^43^. In neutral metacommunities, as the migration rates decrease, beta diversity increases at the cost of alpha diversity^44^. That increase in beta diversity could explain the functional divergence if stochastic processes can override deterministic processes in zooplankton, mosquito, and plant populations. The publicly available datasets that we used in our study, did not allow to test if horizontal transmission affects the conservation of functional composition. To test this, it would be necessary to sample populations with different degrees of horizontal transmission.

An alternate explanation of our results is that only a core subset of functional traits gets preserved across generations. Assuming competition between functionally similar bacteria, Jiang et al.^45^ showed that there is a subset of genes that shapes the structure of a community, denoted as community structure and shaping genes. Given that those genes are carried by a minority of the community^45^, the communities across different generations would not belong to the same attractors. Finally, (microbial) gene composition and community structure do not define function on their own^46^. There could be conservation at a metabolome or gene expression level instead of higher biological levels (i.e. by using KEGG pathways and GO terms). Thus, the actual proteins and/or metabolites produced by the microbiota in a host could be preserved even if the gene composition or the predicted functions of the genes change. This could be tested by using metabolome or gene expression datasets (instead of the KEGG or GO annotations used here) to build the dissimilarity matrices used to find the attractors.

### Ecological succession in the functional space

The initial composition of human microbiota is vertically transmitted, through contact with the mother^33^. Hence, we used human samples to test if that initial composition leads to an ecological succession. Both human datasets showed an ecological succession across the functional phase space, in agreement with the bacterial traits based succession from^47^. This succession can be understood as an extension of the development of the host^46^. This succession was not disrupted by the differing modes of delivery, suggesting that it is robust for a wide array of initial community compositions, which is also true for most traits in the succession from^47^. Similarly, the phylum level composition of the gut microbiota during the first weeks has a trajectory over time that is independent of the delivery mode and is at least partially driven by interphylum interactions^48^. Given that breastfeeding affects microbiota alpha and beta diversity^33^, as well as functional composition^49^, it could be argued that it contributes to the stability of the ecological succession. Since both datasets used here contain breastfed and non-breastfed individuals and all samples still converge to a single attractor at the end, this contribution is not the only stabilizing mechanism. Additionally, the crosstalk between innate immune system, the epithelial layer, and the microbiota control the community composition^13^. In other words, both the resilience and resistance of the community^46^ and host mediated mechanisms could contribute to the stability of the ecological succession.

### Pairwise interactions as a cause of metastability

The SOI simulations did not display metastability, which suggests that pairwise interactions alone do not account for community metastability. The generalized Lotka-Volterra (gLV) models also emphasize first order interspecies interactions^14^, so the analytical results of gLVs should coincide with the results of the SOI model. It has been shown that as community richness increases, the probability of gLVs of having fixed points (community configurations that remain constant over time) decreases exponentially^50^. Moreover, the probability of stability of the fixed points increases asymptotically to one^50^, which means that the communities will be able to reach those points. If instead of drawing parameters for the species within a community they are drawn for a pool of species from a metacommunity, the communities display metastability for specific regions of the parameter space, particularly for large metacommunity species pools^51^. Furthermore, those attractors are non-fixed points, and the community composition is history dependent and can be changed with perturbations^51^. Nevertheless, gLV models can fail to capture the qualitative dynamics of microbiota community when the mechanisms of interaction cannot be represented by additive pairwise effects in the fitness of the populations^52^. Goyal et al.^53^ developed a model that only assumes that each species will only consume one resource at a time and prefers some nutrients over others, and it also displays metastability and transition between local attractors. Experimentally, the fruit fly (*Drosophila melanogaster*) gut microbiota displays metastability, caused by the different colonization strategies of each symbiont (e.g. the preferential attachment to a tissue) and stochasticity^54^. Therefore, metastability and history dependence can arise from pairwise interactions if there is a large metacommunity species pool or with higher order interactions, as with nutrient preference or preferential attachment to a tissue.

### A proposed mechanism for functional recurrence applied to holobionts

**Figure 6:**
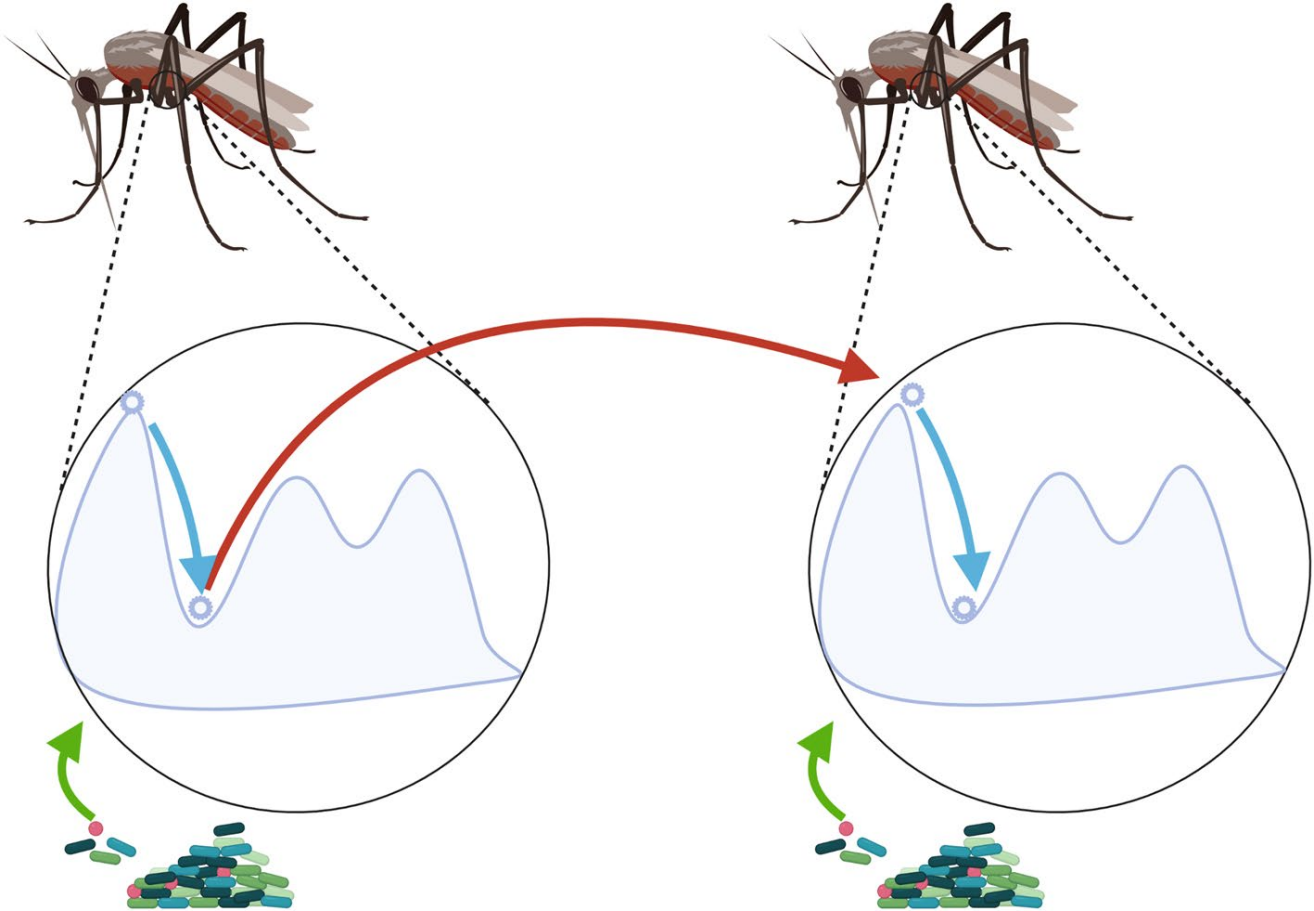
A mechanism for the “It’s the song, not the singer” theory applied to holobionts, created with biorender (https://biorender.com/). The vertically transmitted microbiota initiates an ecological succession in the functional space, which requires the arrival of horizontally transmitted symbionts. The final stable state reached is the same as in the previous generation, which implies the conservation of the functional composition across generations.

Taking all the results together, we propose the following mechanism (Fig. 6) to explain inter-generational functional recurrence^19^. Given a population of holobionts and a rich metacommunity, the vertically inherited fraction of the microbiota defines the initial composition of a host. From that starting composition, there is a robust trajectory in the functional space analogous to an ecological succession, showed in the human datasets. This succession requires horizontal transmission and relies on the (high) richness of the metacommunity, as suggested by the mosquito and plant datasets that lacked those and did not show succession. In our simulations the pairwise interactions alone were insufficient to achieve stability of the trajectory and the mature community, suggesting that this additionally requires higher order interactions between symbionts and with the host. If the trajectory always converges to the same attractor in the functional space, there would be conservation of functional composition across generations. Further experiments are needed to test the complete mechanism, particularly if the predicted gene functions are the adequate level of resolution to look for functional convergence and if there is a robust trajectory in the functional space leading to that convergence for other organisms. The robustness of the functional succession and the final composition need to be tested experimentally. Moreover, there is not yet a model that explains how and when the host could induce or promote the community level metastability and robustness. From an ecological perspective, it remains to be seen if there is a characteristic function-abundance distribution, and if there is, how it could be influenced by stochastic and deterministic assembly processes. From an evolutionary perspective, that function-abundance distribution and the (functional) composition could also be affected by demographic (host population growth and decrease), microevolutionary (host selection, drift, migration and mutation) and holobiont specific (symbiont mutation, horizontal gene transfer, horizontal transmission) processes.

### Implications for animal breeding

The holobiont concept has practical implications for animal breeding^4^. If the functional (instead of the taxonomical) composition is preserved across generations, then the mixed models used in animal breeding could be adapted to account for that, hence potentially increasing the explained phenotypic variance. A similarity matrix between individuals has been calculated based on the taxonomical composition^5,55–57^, therefore, individuals with different bacteria that perform similar functions will still have a high dissimilarity. Nevertheless, if the functional composition is vertically inherited according to the proposed mechanism, then using similarity calculated based on the functional composition could increase the progress over time. Finally, if the community of a given holobiont follows a clear ecological succession, then the optimal moment to sample microbiota composition would be when this equilibrium is reached. The trait turnover in the succession from Guittar et al.^47^ decreased before the taxonomical turnover, suggesting that the functional composition could also reach a mature state earlier than the taxonomical composition.

## Conclusions

The main limitation of considering holobionts as units of selection is that it does it not directly account for the recurrence across generations of horizontally transmitted bacteria, which gave rise to the idea that the functions performed by the symbionts could self-perpetuate over host generations regardless of who is performing the function. In this study we propose that vertically inherited symbionts start an ecological succession that reconstructs the final functional composition of the community, which requires the arrival of horizontally transmitted migrants to be robust. This cannot be explained with pairwise interspecies interactions only, hence host-microbiota interactions and higher order interactions are required to explain the stability of the final community. To establish whether the concept of holobionts as units of selection have a wider applicability, including animal breeding, requires investigating this mechanism for a wide array of host species and applicability.

## Data Availability

All the code for the landscape analysis is available on Github (https://github.com/Christian-Ramos-Uria/Holobiont-Landscape/tree/master/Landscape). All the code for the community simulations is available on Github (https://github.com/Christian-Ramos-Uria/Holobiont-Landscape/tree/master/Beta-diversity). The datasets analysed during the current study are available in the datadryad and ENA repositories, https://www.ebi.ac.uk/ena/browser/view/PRJNA703930 or https://github.com/reillyowencooper/daphnia_multigenerational_antibiotics^31^, https://datadryad.org/stash/dataset/doi:10.5061/dryad.98jj7gk^32^, https://www.ebi.ac.uk/ena/data/search?query=PRJEB20603^11^ , https://www.ebi.ac.uk/ena/data/search?query=PRJEB14529^33^, https://www.ebi.ac.uk/ena/data/search?query=PRJNA290380 or https://www.ebi.ac.uk/ena/browser/view/PRJEB26925^34^.

## Abbreviations

gLV: Generalized lotka volterra
GO: Gene ontology
ITSNTS: It's the song, not the singer (theory)
KEGG: Kyoto encyclopedia of genes and genomes
ASV: Amplicon sequence variant
SOI: Self-organized instability
